# Neuroenhancement of surgeons during robotic suturing

**DOI:** 10.1007/s00464-021-08823-1

**Published:** 2021-11-01

**Authors:** Ronak Patel, Yusuke Suwa, James Kinross, Alexander von Roon, Adam J. Woods, Ara Darzi, Harsimrat Singh, Daniel Richard Leff

**Affiliations:** 1 Deparment of Surgery and Cancer, Imperial College London, London, UK; 2 Department of Clinical and Health Psychology, Center for Cognitive Aging and Memory, McKnight Brain Institute, University of Florida, Gainesville, FL USA

**Keywords:** Surgical training, Robotic surgery, Transcranial direct-current stimulation, Motor skills

## Abstract

**Background:**

The initial phases of robotic surgical skills acquisition are associated with poor technical performance, such as low knot-tensile strength (KTS). Transcranial direct-current stimulation (tDCS) can improve force and accuracy in motor tasks but research in surgery is limited to open and laparoscopic tasks in students. More recently, robotic surgery has gained traction and is now the most common approach for certain procedures (e.g. prostatectomy). Early-phase robotic suturing performance is dependent on prefrontal cortex (PFC) activation, and this study aimed to determine whether performance can be improved with prefrontal tDCS.

**Methods:**

Fifteen surgical residents were randomized to either active then sham tDCS or sham then active tDCS, in two counterbalanced sessions in a double-blind crossover study. Within each session, participants performed a robotic suturing task repeated in three blocks: pre-, intra- and post-tDCS. During the intra-tDCS block, participants were randomized to either active tDCS (2 mA for 15 min) to the PFC or sham tDCS. Primary outcome measures of technical quality included KTS and error scores.

**Results:**

Significantly faster completion times were observed longitudinally, regardless of active (*p* < 0.001) or sham stimulation (*p* < 0.001). KTS was greater following active compared to sham stimulation (median: active = 44.35 N vs. sham = 27.12 N, *p* < 0.001). A significant reduction in error scores from “pre-” to “post-” (*p* = 0.029) were only observed in the active group.

**Conclusion:**

tDCS could reduce error and enhance KTS during robotic suturing and warrants further exploration as an adjunct to robotic surgical training.

**Supplementary Information:**

The online version contains supplementary material available at 10.1007/s00464-021-08823-1.

Despite the well-established technical advantages of robotic surgery [1], the number of cases required to achieve a consistent and acceptable standard of performance is highly variable [2]. The learning curve has multiple phases with the addition of increasingly complex cases [3], whilst early learning is associated with longer operative times and poorer outcomes [4]. Furthermore, the initial phases of learning exacerbate technical deficiencies such as lower knot-tensile strength (KTS) which have been repeatedly observed with knot-tying in robotic surgery [5, 6].

Hands-on training is essential in the acquisition of robotic surgery skills, yet residents are frequently relegated to an observation role [7]. This is reflected in the opinions of surgical residents who, despite agreeing that robotic surgery will play a key role in their future careers, perceive robotic training as inadequate [8]. This is perhaps unsurprising given the relative infancy of robotic surgery and the additional challenge of teaching procedures when remote from the operating table. Furthermore, attending surgeons are frequently on their own learning curve and retain less control when the trainee is at the operating console [9]. Accordingly, various methods have been implemented to help achieve effective robotic surgical training including the use of dry lab and virtual simulators [10] and the development of standardized guidelines to provide quality training and proficiency benchmarks [11]. The Fundamentals of Robotic Surgery (FRS) [12], currently under evaluation, aims to measure a number of motor skills including, for example, millimetre accuracy in suturing and knot-tying under tension. However, guidelines alone may not overcome the aforementioned challenges with reduced robotic training exposure, and additional training methods alongside this could further improve robotic technical skill acquisition.

Transcranial Direct-Current Stimulation (tDCS) is a non-invasive brain stimulation method that involves passing a weak direct electrical current (e.g. 1–2 mA) through two or more electrodes placed on the scalp for a short duration (e.g. 20 min), which can transiently modulate neuronal excitability [13, 14]. Outside the field of surgery, tDCS has improved motor skills, specifically in hand dexterity [15, 16], gross motor skills [17] and limb strength [18–21]. When applied to the surgical setting, multiple studies have demonstrated improved technical skill performance with tDCS [22–26], but these are all restricted to undergraduates which limit their clinical significance. Moreover, the majority have directed stimulation towards motor regions, whereas extensive data suggest that the cognitive phases of surgical skill learning are dependent on the prefrontal cortex (PFC) [27–29]. This brain region is associated with early phases of motor learning where larger variability in motor performance is observed [30]. There is greater capacity for interventions to improve motor skill in this earlier phase of skill development, compared to in experts where ‘ceiling effects’ would limit any potential impact. This is further reflected in prior tDCS research demonstrating significant improvements in lower skilled trainees compared to higher skilled trainees [22, 26].

tDCS applied to the PFC has enhanced task accuracy in finger tapping [16] and golf tasks [17] and also improved performance in multi-tasking [31] and dual cognitive-motor tasks [15]. Recent work has demonstrated significant improvements in surgical open knot-tying skills with prefrontal stimulation [26]. Greater PFC activation during robotic skills has been observed in novices compared to experts [32], but the impact of tDCS in this context remains unexplored. Here we aimed to extend prior tDCS studies by recruiting surgeons instead of medical students, employing a modern surgical platform with a clinically relevant robotic suturing task and finally in line with neuroimaging literature [27–29, 32, 33], stimulating the PFC as opposed to the motor region. We hypothesized improvements in KTS and accuracy in a cohort of surgical residents in the early phases of robotic skills training.

## Methods

### Participants

This relatively novel technique has not been previously investigated in surgeons on a robotic platform and therefore it is challenging to obtain an accurate formal sample size estimate for this experimental paradigm. Instead, a sample size calculation considered prior laparoscopic evidence in students [22, 23] to predict an effect size of a 10% improvement in skill following tDCS versus sham in paired data. To detect a statistically meaningful main effect of stimulation between active and sham groups (α = 0.05) with 85% power, paired data from a sample size of 12 participants were required. Following Research Ethics Committee approval (19/LO/0252), 15 surgical residents (8 males, 7 females; mean age = 33 years, range 28–38 years) affiliated with Imperial College Healthcare Trust were recruited for this crossover study. Residents were recruited via electronic or face-to-face communication and screened for handedness [34], prior surgical experience and contraindications to tDCS. Specifically, participants were excluded if they reported previous robotic surgical experience or any significant neurological history (e.g. traumatic brain injury, stroke, encephalopathy, seizure disorder), history of alcohol and/or substance abuse, psychiatric illness or centrally acting drugs (*n* = 0). Written informed consent was obtained from all participants.

### Experimental design

**Fig. 1:**
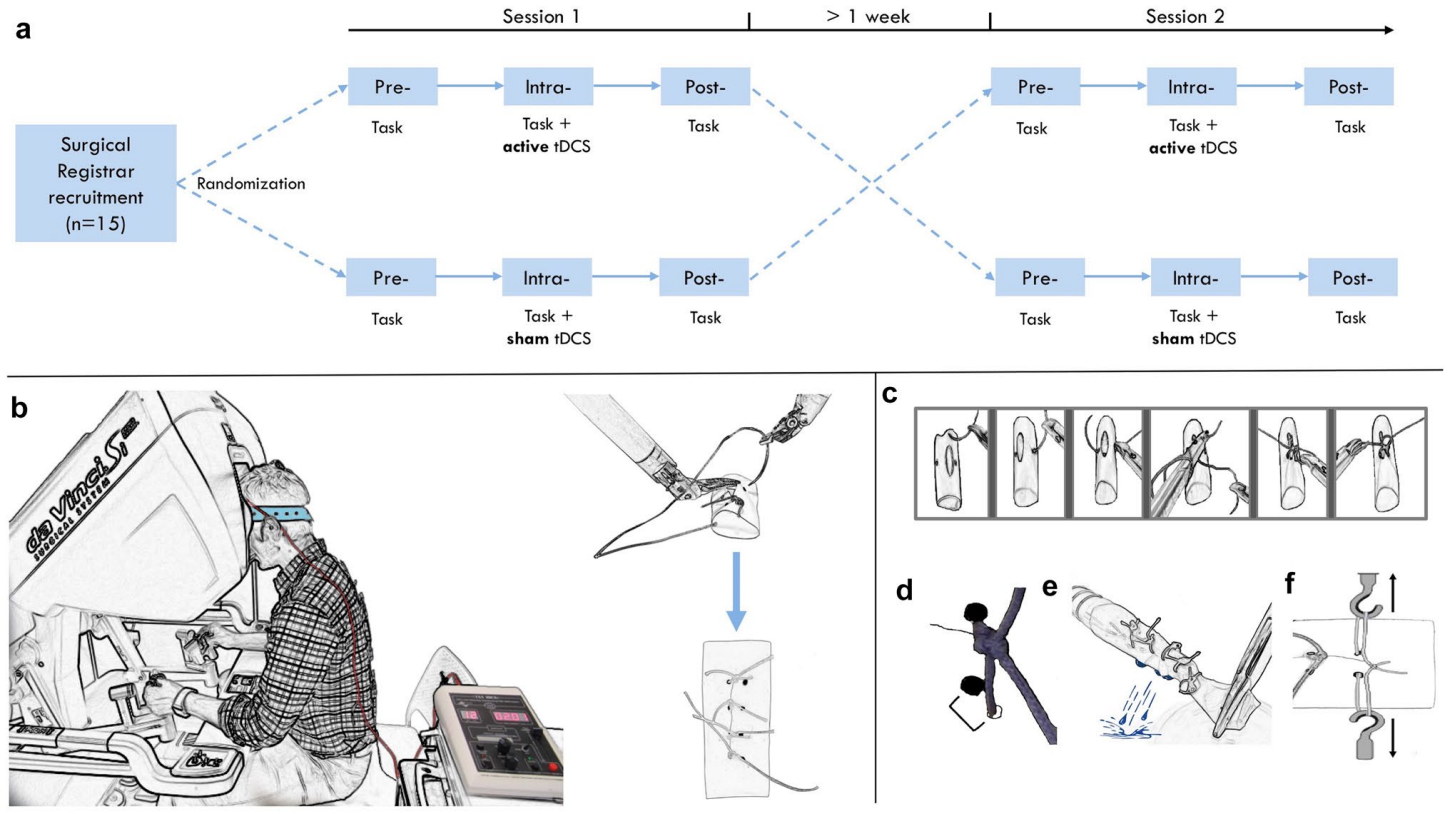
Experimental overview. *Experimental design* (**a**): Participants performed a robotic suturing task three times, which was repeated in a second intervention > 1 week after the initial session. Subjects were randomly assigned to either active (2 mA for 15 min) or sham tDCS and then crossed over. *Robotic suturing task* (**b**): Participant performing task using da Vinci® Si System (Intuitive Surgical Inc., Sunnyvale, California, United States) with concurrent tDCS. The task required securing 4 knots along a Penrose drain at pre-marked entry and exit points. *Technical skill assessment* (**c**–**f**)*:* Progression score (au) **c** with 1 point allocated for successful progression through 6 steps: mounting needle, needle entry, needle exit, double throw, first single throw and second single throw; leak volume (mL) **d** of saline through clamped drain in 1 min; error **e** in distance (mm) from pre-marked entry and exit dots; tensile strength (N) of knots **f** measured using a tensiometer (5565 single-axis tensiometer, Instron, UK)

A randomised double-blind, sham-controlled, crossover design was employed (Fig. 1a). All participants attended two separate sessions, each time receiving a different mode of stimulation (active or sham) at least one week apart to allow for washout of any residual effects of tDCS. The order of stimulation was randomised in a counterbalanced fashion with eight participants receiving active stimulation first and seven participant receiving sham stimulation first. During each session, the participants first underwent 3 min of familiarization with the robotic apparatus. Next, participants performed a robotic surgical suturing task in three separate consecutive blocks. First, a baseline assessment was performed (“pre-”). Subsequently, the task was repeated with concurrent active or sham tDCS (“intra-”). To assess for after-effects, the suturing task was again repeated 10 min after termination of stimulation (“post-”).

### Robotic suturing task

Participants performed a robotic suturing task using an intracorporeal technique (Fig. 1b) on a da Vinci® Si System (Intuitive Surgical Inc., Sunnyvale, California, USA). The task involved inserting a 2–0 Vicryl suture (Ethicon, Somerville, NJ) as close to pre-marked entry and exit points on either side of a defect in a Penrose drain. To tie a knot, participants were instructed to formulate one double throw followed by two single throws of the suture. Within each block, this was repeated four times along the drain, each separated by 30-s episodes of motor rest. Therefore each participant was required to complete exactly 12 knots (4 in each of pre, intra, post) in each session (active or sham), i.e. a total of 24 knots. No additional robotic surgery exposure was experienced between sessions by any participant.

### Transcranial direct-current stimulation

**Fig. 2:**
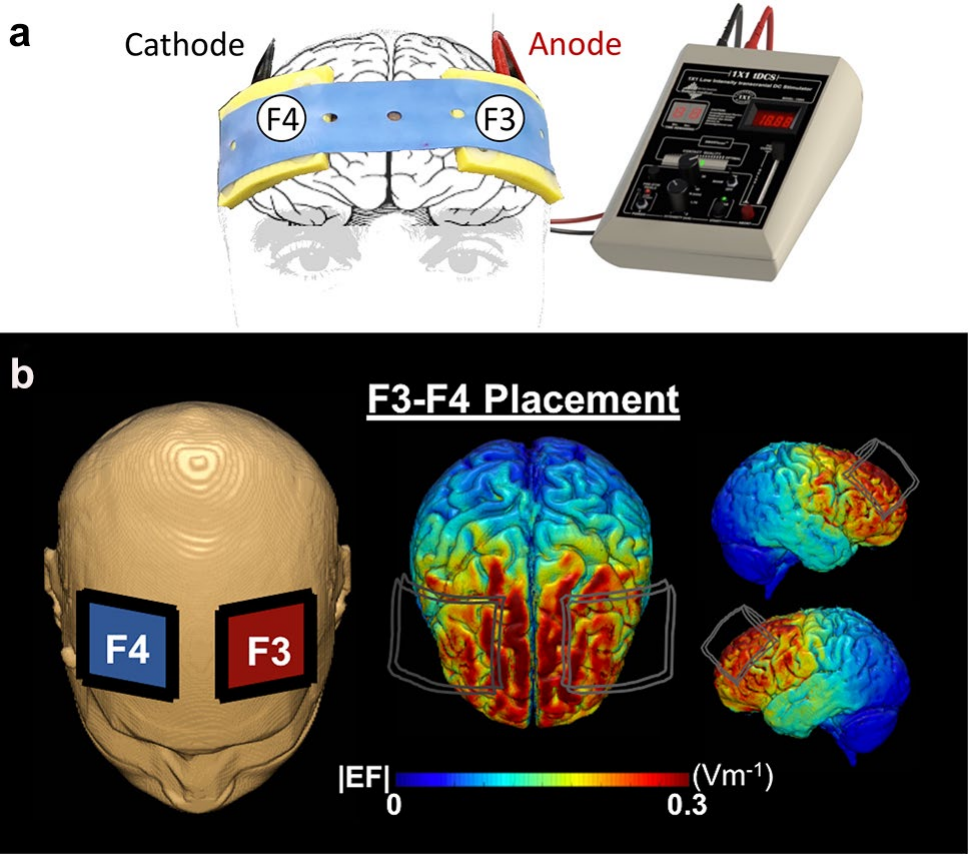
Transcranial direct-current stimulation. tDCS setup (**a**) with red anode and black cathode sponge electrodes placed on scalp and connected to tDCS device to pass 2 mA current through cortical tissue. A computational model (**b**) of electric field distribution for bifrontal electrode arrangement with the anode (red) over F3 and cathode (blue) over F4. The electric field strength and distribution depicted were calculated using a finite element-based approach in ROAST [47]

For bifrontal stimulation a pair of saline-soaked (7mls per electrode) 35cm^2^ (5 × 7 cm) sponge electrodes were affixed to the prefrontal region. Stimulation was delivered using a 1 × 1 tDCS device (Soterix Medical Inc, New York, USA). As illustrated in Fig. 2, the anodal electrode was affixed to left prefrontal cortex (F3 on the 10/20 electrode system) (35) and the cathodal electrode to the right prefrontal cortex (F4). This montage was employed due to the increasing evidence suggesting that 2 mA produces a net increase in excitability under the anode and cathode electrodes [36–38]. Accordingly, we selected this bilateral frontal montage at 2 mA to elicit a net increase in excitability in the prefrontal region, which is further justified by prior studies demonstrating enhanced cognitive behavioural outcome measures [39–43] and increased inter-hemispheric connectivity following stimulation with bifrontal tDCS [44, 45]. Furthermore, as previously demonstrated, this montage has been used to elicit significant improvements in open knot-tying skills [26]. Both stimulation modes involved a 30-s ramp up to 2 mA. During active stimulation, current intensity was sustained at 2 mA for 15 min, followed by a 30-s ramp down. For sham stimulation the ramp up was followed by an immediate ramp down to 0 mA where it remained for the duration of the block (15 min), which has previously demonstrated successful blinding [46]. Here, both the participant and the investigator measuring outcomes were blinded to the mode of stimulation. Following stimulation, participants were assessed for side effects and asked to guesstimate which mode of stimulation (active or sham) they perceived they received.

### Outcome measures

Technical skill was objectively assessed through direct observation of performance and end-product analysis as depicted in Fig. 1 (panels c–f), as used previously [33]. In summary,

- Knot-Tensile Strength (KTS; Newtons, N): A bench-top tensiometer (5565 single-axis tensiometer, Instron, UK) was used to quantify the tensile strength of every tied knot.
- Error Score (mm): calculated as follows = [distance (mm) between needle insertion point and pre-marked target position + distance (mm) between needle exit point and pre-marked target position].
- Time (s): the time-taken to complete each knot
- Task Progression Score (TPS; arbitrary units, au): 1 progression point for each successful progression through the task, including mounting needle, needle entry, needle exit, double throw, first single throw and second single throw (maximum score = 6).
- Leak Volume (mL): The volume of saline leaking from the closed defect over a 1-min period.

Primary outcome measures were KTS and error scores and secondary outcome measures included the time-taken to complete each knot, task progression and leak volume of the Penrose drain.

### Subjective workload

Subjective stress was quantified using the Surgical Task Load Index (SURG-TLX) questionnaire which is a validated measure of introspective workload [48]. SURG-TLX was administered upon surgical task completion. This provided subjective opinions from participants on six domains of workload (mental demand, physical demand, temporal demand, task complexity, situational stress and distractions) throughout each block in both sessions.

### Statistical methods

To assess for carryover effects, a pre-test was performed by calculating the sum of the measured values in each session for each participant and compared across the two sessions by an unpaired *t* test [49]. Following testing of normality using the Shapiro–Wilk test, only leak volume was observed to be parametric, with the remaining outcome variables being non-parametric. Leak volume was analysed using a linear mixed model (LMM) for interaction and main effects of group and block, with participant as a random effect. For the remaining non-parametric outcome measures, performance was analysed using separate generalized linear mixed models (GLMMs) for interaction and main effects of group and block, with participant as a random effect. For the GLMM models, data were transformed where necessary to meet the requirements of a Gamma distribution. This required centring and scaling of the KTS data and inversion of TPS data by subtracting individual score from the highest value in the dataset. Models were compared using the Akaike information criterion (AIC) with the smallest AIC retained. Tukey’s post hoc test was used to correct for multiple pairwise comparisons.

To analyse changes in SURG-TLX scores (non-parametric) over the three blocks, the Friedman’s test was used. The Wilcoxon signed-rank test with Bonferroni correction was used for post hoc comparisons. For comparison of SURG-TLX scores between the intervention groups at each block, the Mann–Whitney *U* test was used. Severity rankings of sensations between the intervention groups was analysed using paired t test and estimation of intervention type was analysed with Fisher’s exact test. A *p* value < 0.05 was considered statistically significant. Analysis was performed using the lme4 package in R v.3.6.3 (The R Foundation for Statistical Computing, Vienna) and SPSS v.25.0 (IBM Corp, Armonk, NY).

## Results

All participants were right-handed and completed both sessions of the study. Outcome measures for each mode of stimulation and block (“pre-”, “intra-”, “post-”) are summarized in Table 1 (surgical performance metrics) and Table 2 (subjective workload measures). No baseline differences in any of the performance measures were identified between the active and sham stimulation groups. Full reporting of statistical analyses is provided in Supplementary Material.

**Table 1:** Performance outcome measures

	Active (*n* = 15)	Sham (*n* = 15)
Time (s)		
Pre	143 (86)	152 (81)
Intra	122 (51)	129 (74)
Post	113 (40)	117 (66)
KTS (N)		
Pre	23.89 (56.10)	30.66 (52.28)
Intra	36.14 (45.72)	31.02 (51.65)
Post	44.35 (32.75)	27.12 (50.64)
Error (au)		
Pre	1 (2)	1 (2)
Intra	1 (2)	1 (1)
Post	1 (1)	1 (2)
Task progression (au)		
Pre	6 (1)	6 (1)
Intra	6 (1)	6 (0)
Post	6 (0)	6 (0)
Leak volume (mL)		
Pre	5.42 (0.86)	5.14 (0.76)
Intra	5.13 (0.80)	5.08 (0.48)
Post	4.83 (0.93)	4.93 (1.00)

Values are medians (interquartile range) except for leak volume (parametric data) which is represented as mean (standard deviation)

**Table 2:** Workload measures

	Active (*n* = 15)	Sham (*n* = 15)	*p* value
Mental demand			
Pre	40 (43)	30 (41)	0.410
Online	14 (37)	22.5 (23)	0.258
Post	16 (18.5)	20 (23)*	0.233
Physical demand			
Pre	8 (15)	7.5 (13.5)	0.861
Online	8 (19)	7 (9)	0.753
Post	5.5 (9)	7 (11.5)	0.972
Temporal demand			
Pre	20 (21)	26 (20)	0.334
Online	8 (15)	15 (18)	0.955
Post	12 (12.5)	13.5 (18)	0.382
Task complexity			
Pre	14 (23.5)	16 (19)	0.944
Online	8.5 (24)	6.5 (20)	0.594
Post	5 (20)*	6 (20)	0.480
Situational stress			
Pre	13 (28.5)	12.5 (19.5)	0.233
Online	17.5 (33.5)	12 (22.5)	0.173
Post	12 (26)	9 (24)	0.221
Distractions			
Pre	0 (3)	0 (1)	0.260
Online	1 (16)	0 (1.5)	0.155
Post	1 (6)	0.5 (5)	0.398

Values are medians (interquartile range)

Asterisk indicates significant difference from the ‘pre-’ block in post hoc testing

*p < 0.05

### Crossover analysis

Statistical analysis revealed no carryover effects for the primary outcome measures (KTS: *p* = 0.898, Error: *p* = 0.895) and the majority of the secondary outcome measures (Leak volume: *p* = 0.661, TPS: *p* = 0.342). Only time exhibited a significant effect (*p* = 0.005) which suggests any results in this domain should be interpreted with caution due to potential for carryover effects.

### Knot-tensile strength

**Fig. 3:**
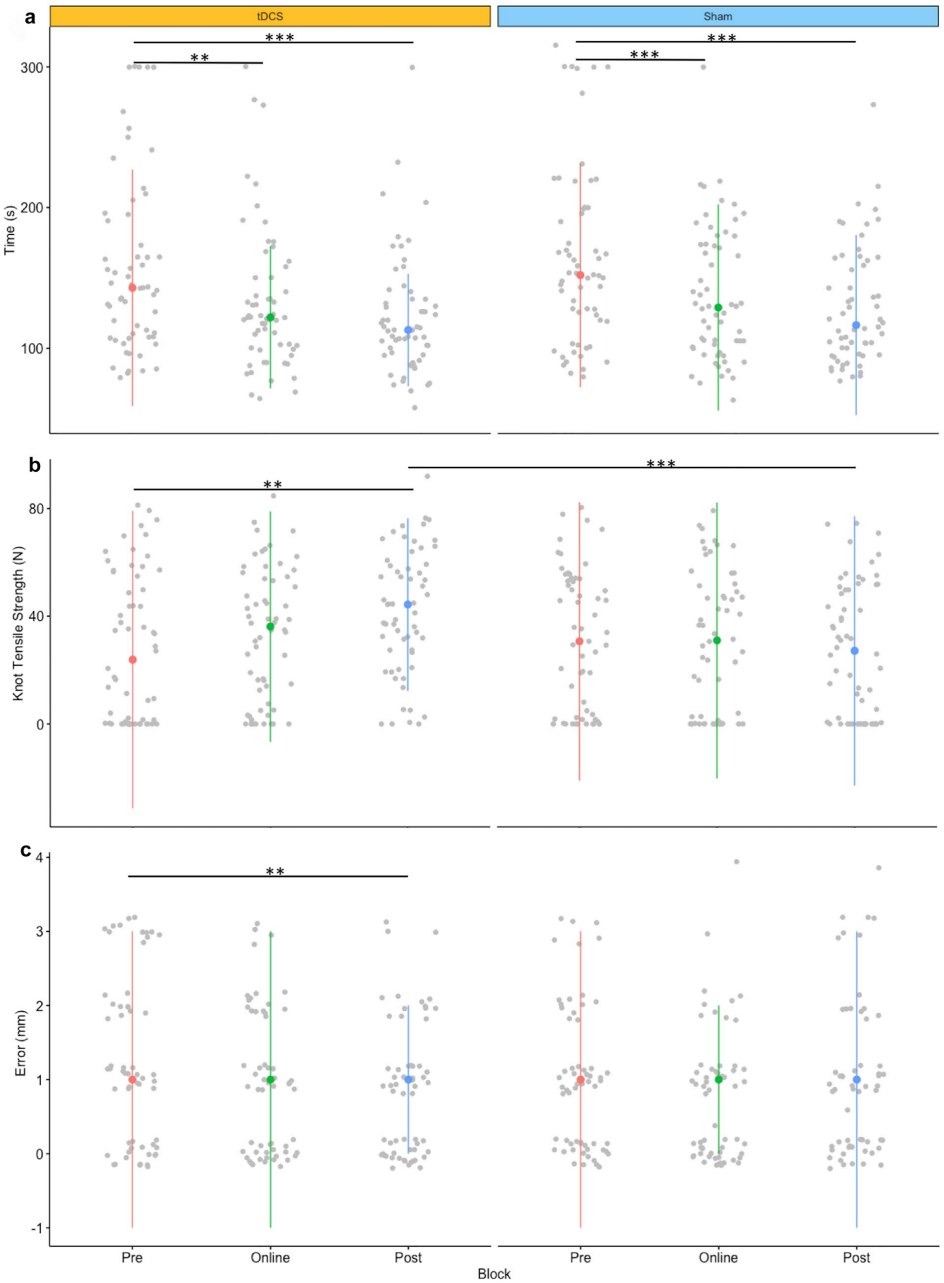
Surgical performance metrics. Scatter plot of individual scores of time (**a**), knot-tensile strength (**b**) and error (**c**) within each intervention group (each knot represented by grey dots). Coloured dots and line represent median scores and interquartile range. Outliers removed to aid graphical representation. Asterisk denotes significant difference, **p < 0.01, ***p < 0.001

The interaction between intervention and block was a predictor for KTS (*t* = − 3.347, *p* < 0.001). As illustrated in Fig. 3, a significant increase in KTS was observed in active stimulation from pre- to post-intervention [median (IQR): pre- = 23.89 N (56.10) to post- = [44.35 N (32.75), *p* = 0.002]. Significant improvements in KTS were not observed with sham stimulation. Indeed, KTS decreased pre- [30.66 N (52.28)] to post-intervention [27.12 N (50.64)]. A statistically significant difference in KTS was identified between active and sham stimulation in the post-intervention block (*p* < 0.001). No other statistically significant differences were observed between the two stimulation modes.

### Error score

The interaction between intervention and block was a predictor for error (*t* = 2.196, *p* = 0.028). As illustrated in Fig. 3, there were significantly less errors from pre- to post- (*p* = 0.029), although median error scores were unchanged, and improvement instead appeared to arise from reduced variability in error scores [median (IQR): pre- 1 mm (2) to post- 1 mm (1)]. No statistical differences in error scores were observed across blocks in the sham stimulation session or between the two modes of stimulation at any timepoint.

### Performance time

Although a significant carryover effect was observed (*p* = 0.005), trends in knot-tying time were similar for both groups and there were no significant differences between the two groups in any block. A main effect of block was observed for the time-taken to complete the task (t = -2.231, *p* = 0.026). Regardless of whether participants received active or sham stimulation, performance time improved from pre-intervention [median (IQR); *active*: pre- 143 s (86) vs. intra- 122 s (51), *p* = 0.001; vs. post- 113 s (40), *p* < 0.001; *sham*: pre- 152 s (81) vs. intra- 129 s (74), *p* < 0.001; vs. post- 117 s (66), *p* < 0.001].

### Task progression score

There were no significant interaction or main effects in Task Progression Scores (*t* = − 0.539, *p* = 0.590). Scores did not vary significantly from pre- to intra- to post-stimulation in the active [median (IQR): pre: 6 au (1), intra: 6 au (1) and post: 6 au (0)] or sham [pre: 6 au (1), intra: 6 au (0) and post: 6 au (0)] group.

### Leak volume

There were no significant interaction or main effects in Leak Volume (*t* = 0.972, *p* = 0.334). Both groups exhibited a non-significant decrease in leak volume across sessions [active: mean (SD) pre- 5.42 ml (0.86), intra- 5.13 ml (0.80), post- 4.83 ml (0.93); sham: pre- 5.14 ml (0.76), intra- 5.08 ml (0.48), post- 4.93 ml (1.00)].

### SURG-TLX

SURG-TLX scores are summarized in Table 2. Introspective task complexity decreased significantly only with active stimulation [*χ*^2^(2) = 9.0742; *p* = 0.011]. Post hoc analysis revealed a significant reduction from pre- to post-tDCS [pre = 14 (23.5) vs. post = 5 (20), *p* = 0.024]. Mental demand significantly reduced across both active [χ^2^(2) = 6.377; *p* = 0.041] and sham stimulation [χ^2^(2) = 8.808; *p* = 0.012]. Regarding active stimulation, a reduction was observed across all timepoints from pre- [40 (43)] to intra- [14 (37; *p* = 0.134] and post- [16 (18.5); *p* = 0.107], but this failed to reach statistical significance at post hoc analysis. In sham stimulation, mental demand was significantly alleviated from pre- to post-tDCS sessions [median (IQR): pre = 30 (41) vs. post-tDCS = 20 (23); *p* = 0.024]. There were no other differences within or between the modes of stimulation across any timepoints.

### Side effects

Side effects and sensation reporting are provided in Table 3. No serious adverse events were recorded. Across a total of 210 side-effect data points, in 163 (78%) no side-effect sensations were reported. 34 (16%) revealed only mild side-effect sensations, with the majority (43%) reporting a sensation of ‘warmth’. It is important to note that 22 (80%) out of the total of 30 sessions residents felt that tDCS (active or sham) had no effect on their performance, whilst the remaining 8 (20%) felt it had only a slight effect. There was no statistical difference in distinguishing between active and sham stimulation (*p* = 0.726), suggesting validity of blinding with the sham setup. Stimulation type was correctly deduced 8 times (27%) with active stimulation and 4 times (13%) with sham stimulation, whilst an additional 8 responses (27%) were incorrect and a further 10 responses (33%) were recorded as ‘don’t know’.

**Table 3:** Sensations reporting

	Proportion of participants		VAS sensation severity ranking		
	Active (*n* = 15)	Sham (*n* = 15)	Active	Sham	*p* value*
Itching	4	3	1.47 (0.92)	1.27 (0.59)	0.189
Pain	1	2	1.20 (0.77)	1.13 (0.35)	0.774
Burning	7	5	1.80 (1.01)	1.53 (0.74)	0.364
Warmth	9	7	1.80 (0.86)	1.53 (0.64)	0.364
Pinching	3	3	1.20 (0.41)	1.20 (0.41)	1.000
Metallic taste	1	0	1.07 (0.26)	1.00 (0.00)	0.334
Fatigue	1	0	1.07 (0.26)	1.00 (0.00)	0.334

Participant reported sensation proportions and mean severity ranking (SD)

*VAS* visual analogue scale

*Paired *t* test

## Discussion

This double-blind randomised crossover trial revealed a transient increase in knot strength and a reduction in robotic suturing errors following tDCS compared to sham. The performance improvement with PFC stimulation is commensurate with previous tDCS studies demonstrating significant improvements in technical skills in surgery [22–26]. However, the current study is the first to explore the impact of PFC-tDCS in a clinically relevant group (surgeons) on a leading surgical robotic platform.

Improvement in technical skill performance observed in the current study could have significant potential in the clinical setting, especially considering the minimal adverse effect profile of tDCS. Specifically, the difference of 17.23 N (equivalent to the force exerted by a 1.7 kg mass at average gravity) in KTS precipitated by tDCS would suggest the formation of stronger and more secure surgical knots. This is evidenced in prior research using Vicryl sutures which has revealed a decrease in knot slippage from 7 to 4 mm when tying load is increased from 20 to 50 N [50]. In all forms of surgery, a suture not held under the correct tension due to loosely tied knots can lead to postoperative bleeding [51, 52], impaired wound healing and increased risk of wound infection [53], both of which increased the likelihood of complications, such as wound dehiscence and incisional hernias [54]. Robotic knot-tying has previously been demonstrated to be of lower tensile strength compared to conventional knot-tying. Muffly et al. [5] demonstrated that 80% of robotically tied knots of polyglactin 910 were untied and were approximately half as strong as conventional hand-tied knots (57 N vs. 112 N). Furthermore, Reynisson et al. [6] observed that although it was possible to robotically tie knots as strong as conventional hand-tied knots, this was only achieved by 1 in 4 surgeons. Although this is theorized to be due to lack of tactile feedback, it is unclear as to why performance is variable amongst surgeons of similar experience. More recently, KTS of robotically tied knots has been observed to significantly deteriorate under time pressure when compared to self-paced conditions [32]. Neurointerventions such as tDCS which appear to enhance KTS may have the potential to offset such reductions in knot strength brought about by temporal stress.

Notably, greater consistency in robotic suturing accuracy was identified with active tDCS, with a higher proportion of surgeons demonstrating greater accuracy (i.e. lower error scores). Although this is a modest finding, accuracy is unquestionably important in surgery and is one of the main factors justifying the development of robotic surgical systems, which enable the surgeon to conduct more precise and controlled surgery. The advance towards high-precision robotic surgery is being realized across new horizons, such as supermicrosurgery [55, 56], where millimetre precision is crucial to successful surgical procedures [57, 58] and the margins of error are increasingly narrow. For example, there is an estimated accuracy requirement in the region of 50 µm in a range of procedures, including vocal cord excision in laryngology, microvascular anastomosis in reconstructive surgery and vasectomy reversal in urological surgery [58]. Accordingly, the FRS curriculum has defined millimetre accuracy as a key outcome metric when assessing performance in robotic tasks [12] and the present study identified that tDCS may improve robotic technical accuracy. Whilst clarification of these findings will be required on tasks in which errors are measured on an even smaller scale, the results are encouraging and imply neuro-adaptive improvement in surgical accuracy.

The findings of the present study are commensurate with extensive tDCS literature investigating strength, accuracy and error measures outside of medicine [15–21, 31]. For example, Frazer et al. [20] demonstrated that anodal motor tDCS significantly increased motor strength compared to sham tDCS (12% vs. 2%). Similarly, they subsequently observed that consecutive days of tDCS improved force in an upper limb motor task [21]. Furthermore, Hendy et al. [19] revealed a significant increase in strength with strength training combined with tDCS, but not following strength training with sham tDCS or tDCS alone. This improvement was also retained in 48-h retention tests suggesting positive and lasting neurophysiological impact [18]. Moreover, recent findings suggest that tDCS applied to the PFC may actually nullify placebo-induced enhancement of motor force [59]. Dampening down of motor placebo effects through our stimulation protocol provides further evidence to support the validity of the increased KTS being attributable to the application of active tDCS. In terms of accuracy and skill, promising findings have also been observed following PFC-tDCS on fine motor skills [16] and more generalized motor tasks [17], as well as cognitive-motor tasks [15, 31].

Regarding secondary outcome measures, the improvement in time-taken observed across both groups would suggest an expected and natural progression of operative speed as participants became more familiar with the task and platform with repeated practice. This could perhaps account for the ordering effects observed in this parameter only, whereby considerable improvement in time-taken due to practice alone would supersede the influence of tDCS. Good progression scores were identified at the start of both sessions with little room for improvement. However, completing the task and doing so quickly does not necessarily equate to better quality which could be inferred from the accuracy and KTS measures. Although leak volume did not improve, it is likely that a more watertight closure would have necessitated a greater number of sutures, which was not permitted within our task paradigm. Additional sutures would reduce gaps in the defect and therefore more influential on leak volume, rather than subjects inserting tighter sutures at the pre-identified marked zones along the drain.

Precisely how stimulation manifests as performance improvements remains a topic of ongoing debate [60] and at a neurophysiological level would be better interrogated using a platform combining tDCS with functional neuroimaging. However, we assume that given PFC activation is critical for early stages of explicit motor learning [27, 28], that manipulation of PFC efficiency may be responsible [61]. Initial phases of motor learning are characterized by slow and variable performance which is highly dependent upon close sensory feedback [62]. This places considerable attentional demands on an individual and is processed by the PFC during early learning [63]. It is conceivable that in the current study, tDCS enhanced PFC efficiency leading to improved surgical performance metrics. In support of this hypothesis, neuroimaging studies have identified an overall reduction in cortical activation during tasks with tDCS stimulation [64, 65]. This is thought to reflect an increase in neural efficiency of synaptic transmission with a reduction in cortical haemodynamic change required for the same level of neural output. The improved neural efficiency within the PFC could conceivably translate into prolonged task-attention which maintains improvements in technical performance and is perhaps reflected in the subjective reduction of task complexity in SURG-TLX data.

PFC stimulation was targeted using a F3/F4 montage with conventional tDCS, which is thought to provide broad stimulation towards the entire frontal lobes. It is conceivable that this facilitated stimulation of multiple nodes within motor learning and motor execution cortical networks. This could further explain the improvement in knot strength observed here, which has previously been investigated with tDCS directed towards the motor cortex rather than the PFC. Furthermore, increasing research has demonstrated the crucial role of current intensity in the pattern of excitability stimulation. Although stimulation at 1 mA has frequently demonstrated increased excitability under the anode with a decrease under the cathode, recent studies have observed that 2 mA stimulation delivers a net increase in excitability under both electrodes [36–38]. Accordingly, 2 mA bifrontal stimulation has also demonstrated improved cognitive behavioural measures [39, 40, 44, 45] and enhanced functional connectivity in left frontal cortices under the cathode [44, 45]. In keeping with these observations, we utilized this tDCS montage during task performance to stimulate a broad cortical region which is critical for high-level task performance.

### Limitations

tDCS appeared to have no bearing on leak volume, requiring confirmation of the clinical impact of performance improvements. Although as previously mentioned, this could be due to the number and location of suture placement, rather than a failure to improve skills. Similarly, knots that are too tight could lead to ischaemia of wound edges but again the correct knot tension would need to be established in a clinical setting first. We accept that complex robotic procedures are multi-faceted and do not just rely on expert performance in one domain (e.g. robotic suturing) alone. Although improving performance in sub-tasks in this way could benefit the procedure as a whole, whether tDCS improves performance across an entire procedure remains unknown. Furthermore, whilst the key independent variable was deployment of active tDCS or sham tDCS, without neuroimaging data it cannot be said for certain what impact stimulation is having at a brain level. For example, the improvement in ‘mental demand’ in the sham group might be evidence of a placebo effect; however, this did not manifest in improved technical performance as per the active group. Although effective blinding provides confidence in tDCS effects, concurrent neuroimaging data would provide further evidence of the impact of tDCS at a neurophysiological level. Finally, to confirm motor learning, long-term follow-up and assessment of skills are required to demonstrate that any improvement is consolidated and repeatable by surgeons.

### Real-world practicality

There is no doubt that the priority for improving surgical training is to ensure increased and earlier robotic exposure for trainees. For novel training adjuncts, such as tDCS, there still remains a clear need for further research before any benefits in surgical skill enhancement are validated. However, should advantages of its use be established in the future, it is interesting to consider how a technology such as tDCS could be incorporated into surgical training alongside the potential ethical implications of doing so [66]. Safety would be the first priority and consistent with existing tDCS literature, including reports from over 30,000 stimulation sessions [67], no serious adverse effects were reported in this study. Reported sensations were largely mild and tolerable, such that no participants felt their surgical performance was even mildly affected. Additionally, there would be a number of practical aspects to consider. For example, most stimulation durations range between 10 and 30 min [68] which could suggest that tDCS would only be suited to practicing skills for a short duration. However, to improve the utility of tDCS, further work is being conducted into expanding the parameter space of tDCS, including the impact of stimulation duration, intensity and repeated exposure on neuroplasticity [38]. Should it be safe to do so, it is likely that tDCS would be best suited as a training adjunct, perhaps on clinical skills courses—available to those who might choose to use it. It would be of utmost importance that trainees retain full autonomy on using tDCS, whilst also being approved by higher regulatory bodies. However, early qualitative data [69] suggests a general acceptance for its use, should safety and efficacy be assured.

## Conclusion

In conclusion, this study suggests the potential to improve knot strength and possibly accuracy in a robotic suturing task, adding to prior evidence that supports its use as an adjunct to improve surgical performance in experimental settings. However, larger studies that incorporate long-term follow-up are required to determine motor retention alongside the precise parameters, participants and tasks that would gain the most performance benefit from neurostimulation. Future studies should combine tDCS with neuroimaging technology to elucidate the neurophysiological impact of stimulation.

## Supplementary Information

Below is the link to the electronic supplementary material.

Supplementary file1 (DOCX 15 kb)
