# Supplementary material for: Neuroenhancement of surgeons during robotic suturing
Source: Surg Endosc. 2021 Nov 1;36(7):4803–14. doi: 10.1007/s00464-021-08823-1 (PMC9160107; doi:10.1007/s00464-021-08823-1)
Supplement: Supplementary file 1 — Supplementary file1 (DOCX 15 kb) [file 464_2021_8823_MOESM1_ESM.docx]

**Supplementary Data –** Model outputs

| lme4 | **Estimate** | **Std. Error** | **t value** | **p-value** |
| --- | --- | --- | --- | --- |
| **KTS** |  |  |  |  |
| Group | 0.7197 | 0.2716 | 2.650 | < 0.001 |
| Block | 0.7672 | 0.2078 | 3.692 | < 0.001 |
| Group:Block | -0.4459 | 0.1283 | -3.347 | < 0.001 |
|  |  |  |  |  |
| **Error** | | | | |
| Group | -0.44152 | 0.18938 | -2.331 | 0.020 |
| Block | -0.34433 | 0.13660 | -2.521 | 0.012 |
| Group:Block | 0.18535 | 0.08442 | 2.196 | 0.028 |
|  |  |  |  |  |
| **Time** | | | | |
| Group | 10.919 | 8.564 | 1.275 | 0.202 |
| Block | -12.813 | 5.742 | -2.231 | 0.026 |
| Group:Block | -1.826 | 3.695 | -0.494 | 0.621 |
|  |  |  |  |  |
| **TPS** |  |  |  |  |
| Group | 0.01469 | 0.11494 | 0.128 | 0.898 |
| Block | 0.04804 | 0.08212 | -0.585 | 0.559 |
| Group:Block | -0.02728 | 0.05064 | -0.539 | 0.590 |
|  |  |  |  |  |
| **Leak Volume** |  |  |  |  |
| Group | -0.4556 | 0.4223 | -1.079 | 0.284 |
| Block | -0.4833 | 0.3091 | -1.564 | 0.122 |
| Group:Block | 0.1900 | 0.1955 | 0.972 | 0.334 |
|  |  |  |  |  |
